# Present(ed) bodies, absent agency: “patients’ perspectives” at the Museum Vrolik of the body and medicine

**DOI:** 10.3389/fsoc.2024.1410240

**Published:** 2025-01-23

**Authors:** Azia Lafleur

**Affiliations:** Museum Vrolik, Amsterdam UMC, Amsterdam, Netherlands

**Keywords:** affect, embodiment, medical museums, illness, disability, narratives, critical health humanities

## Abstract

Medical exhibits are complex spaces, especially when displaying human remains. This research focuses on Amsterdam’s Museum Vrolik, a prominent museum of the body and medicine in the Netherlands with an important role in the conservation and exhibition of the material heritage of Dutch medicine of the 18th and 19th centuries. I am interested in the affective encounters that are at play in such a setting between us—the living—and the remains on display: How the agency and subject-hood of those who lived and live with ill health, medicalization and disability are effectively present and absent in the context of affective influences in the Museum Vrolik. I deploy the concept of “patients’ perspectives” as a conceptual tool for looking at those who have been impacted by medicine’s medicalizing gaze and handling. Their presence/absence is investigated by using embodied inquiry to attend to the affective encounter between the audience and the bodily remains on display, as felt through the embodied experiencing of visiting the exhibit and mediated by the cultural, physical and institutional context and curation of the Vrolik itself. To analyze the resulting data, I take the museum as a site of storytelling with its curatorial techniques and texts acting as narratological frames and “orientation devices”. The most central pattern emerged as a dissonance between the affective orientation I bring into the space due to my own situated-ness and the orientations prompted by the museum’s frames. The remains on display have been decontextualized from their original home as a part of someone, and transformed into “specimens”. At the same time, my lived experience and identity as a person with chronic illness brought an impulse/intensity towards identification and closeness to the “specimens”, grasping for a sense of their agency, voices, perspectives, personhood. To move forwards from here, persons with disabilities, illness, bodily differences, impairment and injury need to be included and recognized in their capacity as knowers, as having vital embodied knowledge via their lived experience, as narrators and subjects in the stories that are told.

## 1 Introduction

Medical exhibits are complex spaces, especially when displaying human remains. This research focuses on Amsterdam’s Museum Vrolik, a prominent museum of the body and medicine in the Netherlands with an important role in the conservation and exhibition of the material heritage of Dutch medicine of the 18th and 19th centuries. Originally based on the anatomical collection of physicians Gerard (1775–1859) and Willem (1801–1863) Vrolik, it is now located on university hospital premises. While it still fulfills some of its original function as a site for medical education, over time it has become self-aware of its role as preserver of material heritage and seeks to respond to a broader public interest in the history of medicine. It has also begun engaging with issues surrounding the collection and display of human remains—particularly by investigating the colonial past of some of the collection. With the Vrolik’s main display still consisting mainly of historical (largely human) specimens, it highlights some of the key tensions inherent in medical museums: Navigating a collection with problematic origins, seeking to stay relevant as a site for research and education, while also trying to welcome broader audiences and responding to contemporary debates on health and heritage (Arnold, 2004).

As a researcher, audience member and chronically ill person, I am interested in the affective encounters that are at play in such a setting between us—the living—and the remains on display. Following Porter’s (1985) epistemic critique of medical historiography overlooking the “patient’s view”, my research focuses on *how the agency and subject-hood of those who lived and live with ill health, medicalization and disability are effectively present and absent in the context of affective influences in the Museum Vrolik*. I do this by examining how our encounters in the Vrolik move us and are mediated, giving us an “orientation” (Ahmed, 2006) towards seeing through certain eyes by “foregrounding” and evoking empathy and identification with certain perspectives rather than others. This research builds on a phenomenological interest of prioritizing lived embodied and sensory experiencing, as well as a concern with the “liveliness of matter” (Truman, 2019, p. 2) as centered in feminist new materialisms. To fundamentally incorporate the understanding of knowledge as corporeal and situated, embodied experiencing becomes a source of data in the act of empirical research. This led to using the method of embodied inquiry (Brown and Leigh, 2021) allowing me to draw on my own embodied and sensorial experiencing in the museum as data for analyzing its affective, emotive, visceral and empathetic entanglements. This meant exploring how I am “placed” and encouraged or discouraged to place myself in relation to the objects/specimens/bodies in the exhibit via the mediating practices of collecting, preparation, curation, presentation and narration in the museum context. These mediating practices and the affective data generated through embodied inquiry are further interpreted as stories. Analyzing the data with narratological tools allows me to untangle the museal encounter as a co-authored experience between the audience, the curators, the displayed remains themselves and the historical anatomists. This enables me to search for “patients’ perspectives” by examining the mediating “frames” that “orient” us towards particular “perspectives”, or “points of view”, thereby making sense of my affective responses.

This research can be situated in the field of medical and health humanities which emphasizes the agency, subject-hood and essential role of those experiencing illness, disease, disability, impairment and medical treatment. The humanities have also been credited with impacting the practice of modern anatomy towards a more humanist approach (Štrkalj, 2014). Moreover, academic and scholarly sidelining of these experiences has also been increasingly addressed and counterbalanced in the practice of medical historiography (Stolberg, 2011), disability studies, activist history, mad studies, “crip theory”, critical health humanities, and innovative projects in the history of medicine, illness and disability (Davies et al., 2021). One approach involves the collection and display of innovative source material, such as recordings of psychotherapeutic sessions, private personal effects, journals and autobiographical material of ill or disabled persons of the past (Birdsall et al., 2015; Davies et al., 2021; Scarfone, 2020). However, many such archival projects prioritize not only the gathering and writing of history *about* the ill and disabled, but actively collaborate on such research and writing *with* them, as seen in projects such as DisPLACE (2022), After the Asylum (2019) and History in Practice (Davies, 2014). Furthermore, many scholars are engaging with topics of health, illness, disability, healing, medicine and history with the insight of their own lived experiences (Brown and Leigh, 2020; Toombs, 1992). This larger shift is also taking place in the case of museum exhibitions, such as “Bedlam: The Asylum and Beyond” (Harris, 2017) which incorporated ill persons’ narratives as well as their artwork and reflections; “Misbehaving Bodies: Jo Spence and Oreet Ashery” in which the artists contemplated their own experiences of care and illness (Vasey, 2020); and “Medicine and Treatment” which included the sharing of personal experiences and stories of being on the receiving end of medical treatment (Bond et al., 2021). These examples illustrate the shift away from an exclusively medicine- and doctor-centric view, towards centering those who were and are experiencing illness, disability, and practices of healing and medical treatment. Throughout this research, these strategies and approaches served as reference points and helped broaden my perspective for what is possible and achievable in the context of a “medical museum”.

The Museum Vrolik itself has already been concerned for some time with many of the issues raised in this paper, is engaging in research on several of them and seeks to change the exhibit in the near future to actively include more marginalized perspectives. Here, I hope that my critique can serve to highlight affective and empathetic responses in addition to cognitive engagement. To acknowledge specimens as not merely transparent vehicles for (anatomical) knowledge renders them more resistant to classification and objectification, freeing them from exclusively scientific frames. At the same time, persons with disabilities, illness, bodily differences, impairment would also need to be included and recognized in their capacity as knowers, as having vital embodied knowledge and epistemic authority, and thus be an explicit part of such a transformation process. This article is thus a starting point for working with the Vrolik to develop new (narratological) framings and curatorial practices with the potential of dismantling common hierarchies embedded in the production of knowledge, and contributing to making the experiences of historically “othered” groups more present.

## 2 The Museum Vrolik case study: from cabinets of curiosities to museums of medicine

The chosen case study—the Vrolik—is situated in a broader history of medical museums in Europe. Its practices, both historical and contemporary, are in conversation with others in the Netherlands and beyond. The origins of contemporary European medical museums can be traced back to the Renaissance and early Modernity, when “medical men” began to accumulate their own collections of “curiosities” and “materia medica” in their workplaces and homes (Arnold, 2004, p. 146). These were sites of research and experimentation, and over time, the collected materials became an integral part of medical education and training, which gave birth to many medical collections attached to medical educational institutions. Such collections were not merely “neutral” sites of education and research, but were entangled in evolving cultural and sociopolitical histories. They emerged at a time when the body was seen as uncharted territory, awaiting exploration and discovery via scientific inquiry and dissection (Sawday, 1995). To be delineated, named, and categorized: “Like the Columbian explorers, these early discoverers dotted their names, like place-names on a map, over the terrain which they encountered” (Sawday, 1995, p. 23): the Fallopian tubes, the Eustachian tube, the pouch of Douglas. Thus, the body in pieces, embellished by the craftsmanship of dissection and preparations of conservation and display, found itself behind glass or on pedestals as trophies or treasures, along with botanical, mineral or other natural matter. “The quantity and diversity of specimens assembled inside these “cabinets of curiosities” became a symbol of status for their owners” (Davidson, 2021, p. 79), demonstrating one’s culture, wealth, travels, and access in the emerging and burgeoning fields of natural history and natural philosophy. In many cases the human remains and objects that were gathered, studied and used formed part of European imperial and colonial projects. In the case of human remains these often acted as material evidence supporting theories of racial difference and reinforcing racist and ableist ideals. This was also the case for Museum Vrolik, where about 8% of the human remains came from the colonial context (de Rooy, 2023).

Museum Vrolik is based on the collections of the anatomists and physicians, father and son, Gerard (1775–1859) and Willem (1801–1863) Vrolik (de Rooy and Van den Bogaard, 2009). As scientists, collectors and preservers, the Vroliks kept their original collection at their home in Amsterdam. After Willem’s death, it was bought and then donated to what is now the University of Amsterdam, and since the 1980’s it can be found as part of the Academic Medical Centre, which includes the university hospital affiliated with Amsterdam university. Until the 1950’s, while being used as a medical laboratory, many successive anatomists of the university contributed to the collection (de Rooy and Van den Bogaard, 2009). Currently the museum “takes care of about 25,000 objects. The permanent exhibition comprises over 2,000 of these objects” (Visit the Museum, 2024). Over the course of the 1990s, the teratological specimens were cataloged (Oostra, 2009) and a series of articles was published in the “American Journal of Medical Genetics” reevaluating the specimens with congenital anomalies from a contemporary genetic and medical perspective (Moorman, 2009), reinstating the collection’s contemporary research value. Presenting itself as a “historical museum of the human body”, the Vrolik prides itself mainly on its human (and to some extent its other animal) anatomical preparations, consisting of “wax models, plaster models, anatomical preparations in liquid, dried anatomical preparations injected with wax and dried skeletons and skulls” (About the Museum, 2024). Although not found on display, the museum also contains in its archive: glass slides and photographic negatives, antique medical objects, tools and instruments of Amsterdam hospitals and the medical faculty, as well as materials of dentistry and botany (Collections, 2024). As with other collections, a lot of the animal specimens had been split from the original collection. Many of these are now back on display at the Vrolik as a loan from Naturalis Biodiversity Center in Leiden to better represent the collection’s historical makeup.

In 2012 the Vrolik reopened after a major restructuring of its permanent exhibit, with the intention of making the exhibit more accessible and engaging to a wider audience than the medical and scientific researchers and students that had been its main audience (de Rooy and Moorman, 2011). At the time of my visits over the spring and summer of 2022, the museum was still in process of creating its identification guides, which name and explain all the specimens and objects on display, following its declared intention for accessibility to a broader lay audience. The Vrolik’s main display still consists almost exclusively of historical specimens, making it both a typical medical collection that is engaged with current debates and yet choosing different modes of engagement from other institutions of its kind.

## 3 Theoretical framework

### 3.1 Patients’ perspectives

British historian Porter’s (1985) essay, *The Patient’s View: Doing Medical History from Below*, is a critique of the conventional, physician-centered historiography of medicine. Porter advocates an alternative, pluralist account of the history of medicine, one that fundamentally includes the “patient’s view”, with the ultimate goal of broadening the field towards a history of healing, health and illness. His efforts towards building this “history from below” start with outlining the historical misrepresentations involved in the “implicitly endorsed […] view that the history of healing is par excellence the history of doctors” (Porter, 1985, p. 175). The medical encounter is an (at least) two-person affair of the doctor and “patient”. Medicine as a field of scientific knowledge and practice owes its existence to patients’ health and sickness and to their material bodies for research and treatment. Porter suggests that the medical establishment produces “histories of itself essentially cast in the mold of its own current image” (Porter, 1985, p. 175). This re-frames the telling of history as something beyond the account of what occurred, and highlights the bias involved. This informs my current project by pointing to a gap in institutional knowledge and by encouraging me to actively search for “patients’ perspectives” with an attentiveness not only towards what is present (ed), but also towards what is absent. This includes other concerns, beliefs and practices around health than those included in physician-centered histories of medicine. Porter notes the example of how health was a communal concern rather than an individual matter confined to institutionalized or medicalized roles. Furthermore, taking the diversity of experiences, practices and forms of knowledge about health and healing into account can also serve to humanize the establishment of medicine itself as consisting of people, themselves vulnerable to illness, disability or injury, in mind and body.

Despite the theoretical and historical importance of Porter’s essay, at times his approach to the “history from below” lacks intersectionality. His claim that “pain has been even-handed enough to visit the rich, educated, and visible scarcely less than the poor” (1985, p. 183) overlooks the immense specificity of the experiences of ill-health based on people’s literacy, education, class, and social, ethnic and gender identities. While anyone can fall ill, those who are in precarious socio-economic positions, people of color and people of marginalized identities are disproportionately more likely to experience ill-health, as well as complicated and often negative encounters with medical professionals and difficulty in accessing treatment (Epstein, 2007). Everyone can fall ill, however there are plenty of illnesses that only occur in those who have uteruses and for which medicine still grapples with addressing. Anyone could be or become disabled, but if you have the means and social capital to receive care, assistance and access, then living with disability will look radically different.

A history of medicine/health that does not consider these intersections fails to truly be a history from the actual diversity of “patients’ perspectives” and falls into issues similar to those Porter tries to criticize. If we fully consider the implications of gathering overlooked histories, of those who were excluded from the master narratives of medical history, then it must be intersectional. The many histories of the ill, of the disabled, of the neurodiverse, of marginalized genders, sex, ethnicities and socioeconomic classes are not separate nor mutually exclusive, and taking this into account can only enrich our collective understanding, nuance and (situated) knowledge (Haraway, 1988). Taking an intersectional approach that inquires into the dynamics of social power of the past is not about “castigat(ing) the sexism, racism, and other-isms of our forebears” (Bynum, 2008, p. 4) as some medical historians complain. It is about taking a critical eye towards those whose voices were or are idolized in contrast to those whose voices were excluded from the public discourse or production of authoritative knowledge and who’s perspectives take dedicated work to bring to light today. It means including an awareness and a questioning of these very dynamics of power and oppression into our historiographical processes. To mark this conceptual shift, I employ the plural “patients’ perspectives” over Porter’s singular “patient’s view”.

### 3.2 A note on language and terminology

The term “patients’ perspectives” is not the most applicable when we wish to center the diverse perspectives of those experiencing illness, disability, impairment, injury, etc. Using the word “patient” places ill and disabled people into exclusively medical terms, and medicalizes those who may not be or see themselves as patients. It also overshadows those who are undiagnosed, or struggle to even access the status of “patient”. Furthermore, it reinforces the false doctor-patient binary, wherein doctors are not seen as beings who experience health and ill-health within their own bodies, as well as the dichotomy between health and illness/disability, which are not mutually exclusive categories. Moreover, illness and disability can be both overlapping or entirely separate experiences (Wendell, 2001), and one can experience differing health or ill-health on multiple dimensions, be it mental, physical, emotional or social. On an existential level, health, illness, pain, healing and medicine are ubiquitous, universal to the human experience. And yet, when being ill, chronically ill, injured or disabled forms a defining part of one’s life, these experiences are immensely specific and fall outside of dominant norms and expectations.

For the purpose of this research, I nonetheless deploy the term “patients’ perspectives” as a conceptual tool for looking at those who have been medicalized by virtue of their bodies being handled and treated by medical practitioners, whether in life or only posthumously, and whose remains are the objects of the medical museum in question. It is also worth noting that many of these bodies were not necessarily patients of the doctors or scientists who made use of their remains. These were acquired post-mortem, and may or may not have had a direct connection to the medical practitioners themselves prior to their death. As such, “patient’s perspectives” serves as a conceptual tool that holds a diversity of perspectives within it, defined in this particular research by their being on the receiving end of a process of medicalization and medical objectification.

### 3.3 Affective encounters

The second foundational impetus of this research is a phenomenological interest in centering lived, embodied, and sensory experiencing as sources of knowledge and meaning-making. On the one hand, this serves to elevate the epistemic authority and value of those with illness and disabilities as “knowers” in matters of health, illness, disability and the body. This applies to the present as well as the past, thereby asserting their crucial role in the history and historiography of health and medicine. On the other hand, it also informs the theoretical and analytical approaches towards searching for “patients’ perspectives”. Their presence/absence is investigated by attending to the affective encounter between the audience and the bodily remains on display, as felt through the embodied experiencing of visiting the exhibit and mediated by the cultural, physical and institutional context and curation of the Vrolik itself.

By examining affective encounters between bodies, I prioritize the forces and intensities that move them, that impact and transform them, that affect their becoming (Truran, 2022). Affect does not quite belong to one body or another, but rather “it emerges from encounters between them that impede or facilitate either’s ability to act, *to be*” (Ingraham, 2023, p. 3). Through these encounters we find and situate an affective realm involving all body-entities as well as the space itself—animating even seemingly inert materiality with a “liveliness of matter” (Truman, 2019). Following feminist new materialisms, materiality “is always more than “mere” matter: an excess, force, vitality, relationality, or difference that renders matter active, self-creative, productive, unpredictable” (Coole and Frost, 2010, p. 9). This conceptualization enables me to approach the bodies on display with an acknowledgement of their potentiality for agency, action and animacy; for what they can do, be and become; thereby blurring the boundaries between bodies as subjects and objects.

In her work on emotions, Sara Ahmed “connects lived experience, emotion and affective contact” (Truran, 2022, p. 29) by conceptualizing how “we are affected by “what” we come into contact with” (Ahmed, 2006, p.2) and how emotions “create the very effect of the surfaces or boundaries of bodies and worlds” (Ahmed, 2004, p. 117). Using the phenomenological concept of orientation, she highlights how emotions occur in the “contact” between bodies and thereby also shape how we approach, face, move and “turn” “towards” or “away” from them. She especially attends to how histories shape how we arrive to an encounter, how we “place” ourselves and are “placed” in relation to other bodies/objects. “Concepts, ideas, attitudes, are “sticky” with emotions and affects, so that we inherit or incorporate ideas that are not fully conscious and not our own” (Truran, 2022, p. 30). In this sense, emotions gather and “stick” to certain bodies/objects/subjects in an accumulation of instances, therefore influencing and being influenced by the social, collective and political. In this way, history and historiography play a vital part in mediating our present encounters: “it matters *how* we arrive at the places we do” (Ahmed, 2006, p. 2). In the context of the Vrolik, this allows me to attend to how I arrive at the museum, as well as how the context of the museum gives orientation to my affective encounters within it.

## 4 Methodology

This section explains how this research uses Embodied Inquiry to move from the affective encounter to creating usable data, which can be patterned and analyzed. The resulting data consists of my observed embodied experiences and rich descriptions of the exhibit, the textual material provided in the exhibit and museum website, and the historical and institutional context surrounding the exhibit. This is all analyzed via a narratological framework that takes museums as sites of story-telling and stories as essential human vehicles for meaning and interpretation.

### 4.1 Data gathering: embodied inquiry

“Embodied inquiry” as a methodological framework for data generation is outlined by Brown and Leigh (2020) in their work *Embodied Inquiry: Research Methods*. It sees the body as an essential part of data collection and analysis, while being combinable with other methods. The Vrolik is a space that is filled with bodies, fragments of human remains, or objects and preparations made to represent body pieces and parts; all that lies inside comes from or aims to represent the body, whether human or other animal. Therein, live bodies of the audience move around and gaze at the bodies on display: They experience an encounter, and subsequently engage in dialogue with or reading/interpreting the exhibit—mediated by the supplemental textual and spatial information provided. Embodied inquiry takes the researcher’s body in the field and in interaction with its context and the other bodies present as a form of investigation and a method for generating meaningful data. Therefore, we can understand the Vrolik as a site of interaction in which meaning can be generated via the information gathered through the embodied responses of being part of the audience in this affective encounter, making my, the researcher’s body, its senses and sensations, part of the material to analyze. This methodological approach follows Feminist New Materialist thought in acknowledging how “the researcher is part of the apparatus that produces the phenomena or event; they are entangled in the research events they create” (Truman, 2019, p. 4). Furthermore, it takes seriously Ahmed’s claim that “knowledge cannot be separated from the bodily world of feeling and sensation; knowledge is bound up with what makes us sweat, shudder, tremble, all those feelings that are crucially felt on the bodily surface, the skin surface where we touch and are touched by the world” (Ahmed, 2014, p. 171). This embodied data was continuously translated into field-notes throughout my data-gathering visits. The field notes consisted of rich descriptions of the exhibit, the space of the museum and the matter within, stream of consciousness observations and reflections, attempts to simultaneously weave in internal and external stimuli, and contextualization in relation to excerpts of the exhibit texts.

### 4.2 Data analysis: mediation and stories

In analyzing the data generated by embodied inquiry, I have to attend to acts of mediation: Firstly, mediation of affect via senses, feelings and emotions, and secondly, the mediation of the encounter between the audience’s bodies and the bodies on display via the context of the exhibit. Thinkers such as Massumi (drawing upon Spinoza & Deleuze) theorize affect as non-verbal, extralinguistic, noncognitive and nonconscious, always in movement and unfolding (Ingraham, 2023; Truran, 2022). As soon as it is cognitively interpreted, emotively defined and linguistically expressed, it ceases to be affect as it becomes “personal” and loses its undefinable excess and immediacy. In this sense, affect theory holds the potential to “force us to think about mediation” (Dernikos, 2020, p. 248) since affect itself escapes the confines of thought. I wish to address the issue of writing about the unlanguageable in this research by explicitly outlining how I apply my own interpretive filtering that is my embodied consciousness to the affective encounter. I do this in order to observe and subsequently verbalize how I relate and feel moved and affected by the bodies on display as well as by the mediating forces of the Vrolik as space and curator/narrator. This may no longer refer to affect in some of its theoretical senses, but to the material effects of affect that I am able to “read” and “express”. In other words, placing this research in a broader conversation on affect, what I analyze is not the force of affect itself, but rather subjectively observable force-effects.

To attend to the mediation of affect via a researcher’s embodied experience (and generate data from it), embodied inquiry depends on developing awareness, sensitivity and reflectiveness to one’s own experiencing and positionality, generating insight into a phenomenon while situating it in the context of one’s embodied socio-cultural position (Brown and Leigh, 2020). While no single experience can be universally generalized, it does add to, enrich and nuance the collective knowledge produced from various epistemic positions (Haraway, 1988). In the case of the current inquiry, my role as researcher is shaped by my experiences with chronic pain and illness which can be often and unpredictably disabling, as well as my role as a patient subjected to the medical gaze. In the practice of “data-generating/gathering”, this aspect of my life leads me to affectively identify with, empathize, relate and be attentive to “patients’ perspectives”. In other words, it gives me an *a priori orientation towards* those whose bodies are exhibited at the Vrolik before I even enter the space. It also shapes my sensorial and physical engagement with the space, for example how much input I can process at a given time or how my body moves around the space. Conversely, this specificity also brings with it a degree of ignorance, on an embodied experiential level, of other forms of physical/mental impairment and living with more visible disabilities and bodily differences, which in turn shapes and limits my insight into lived aspects of such forms of disability and the degree to which I can interpret the exhibit from that vantage point. This is particularly relevant in the context of the Vrolik given the importance placed on vision and on making illness/abnormality *visible*, as well as with the focus placed on human remains that can illustrate physical “anomalies” and “deformities”.

Thus, my particular orientation and position generates data that is both specific and insightful with regard to how the exhibit produces affective force-effects. This data can be analyzed to get at the second layer of mediation: that of the encounter itself. The ways the material of the exhibit is preserved, selected, arranged, displayed, lit, framed and placed in relation to each other; the information given by websites, books, information cards, brochures, walls and tour guides and how they refer to people and objects; the images, furnishings, paint, and layout of the space—all these act as “orientation devices”, ways in which the museum guides the experiences of the audience. In order to approach this layer on the basis of my field notes, I am taking the museum as a whole as a site of storytelling with its curatorial techniques and texts acting as narratological frames and acts. This is because stories and narratives are essential forms of meaning-making (Bedford, 2001; De Fina and Georgakopoulou, 2015) and are fundamental tools for wording/mediating embodied experiences and sharing them with others. Stories “open up a space into which the listener’s own thoughts, feelings, and memories can flow and expand” (Bedford, 2001, p.29) and so we (and our bodies) become the site for the emotional affect of the story to exist. It is in our emotions, our being moved, that the stories’ embodied impact takes place. We become part of this performance of storytelling, bringing in our own point of view, engaging with the values and assumptions embedded in the narration. This is especially true in the museum setting, where audiences co-author the experience by how we choose to move through the space and engage with the information made available.

This approach follows recent impulses as part of the “narrative turn” in the study and practice of museums, which treat exhibits as texts to be read and analyzed in terms of the stories/myths/narrative strategies they produce and employ (Mason, 2006; Parker, 2013). Every piece of the exhibit, every preparation, every aspect of the museum, holds the potential for multiple stories of the different stakeholders involved. Looking at these different perspectives enables us to examine the relationships, hierarchies and value systems implicit within them. Hereby I make use of Niederhoff’s definition of “points of view” as “the way the representation of the story is influenced by the position, personality and values of the narrator, the characters and, possibly, other more hypothetical entities in the story-world” (2014, p. 692). Most centrally, perspective refers to and results from the relationship between the teller, or “viewing subject”, and the told, “a viewed object” (Niederhoff, 2014, p. 694). What and who is “placed” in the position of viewing subject and in the role of viewed object in the (hi)stories of medicine shows which modes of engagement with health are valorized or marginalized, which perspectives are seen as worth preserving and replicating and which are left unaccounted for, who is present as agents and who is reduced to passive roles, who gets to tell their stories and have them heard, and whose stories are absent. Therefore, this research inquires to what extent “patients” are cast in the role of agential subject, enabled to tell their own stories from their perspectives. How, in other words, we as audience are oriented in such a way as to perceive their (potential) animacy and agency.

## 5 Analysis: a multiplicity of stories

### 5.1 Arriving in the Vrolik: affectively experiencing dissonance

Edited excerpt from field notes:

Walking into the large single room that makes up the Vrolik museum, the sudden quietness and the darkness cut by beams of light from angled spotlights through the rows and rows of cabinets are immediately impressing my senses. From floor to walls to ceilings, everything solid is painted in a matte black. The only sounds are those of visitors murmuring to each other, the venting air from above, and footsteps and the rustle of clothing as people move around. I cannot decipher what I smell; it feels neutral, a bit stale, enclosed. It also smells a bit old, like a second-hand shop or a library… that’s probably the old wood. The cabinets are mostly glass prisms, but there are also many antique-looking and embellished wooden ones, what I imagined typical “cabinets of curiosities” to look like. They are all completely packed with anatomical specimens, skulls, bones, and some models and casts of different materials, I am guessing wax or plaster, but it is hard to tell with my untrained eye. The items inside tend to be of a faded yellow, cream, white color; with some reds, browns, and darker colors in the mix—but all in unsaturated aged hues. The lights from above and inside the ceiling of the cabinets shine a warm yellow glow. The spotlights in the darkness give the specimens a majestic quality.

The effect feels like being in a time capsule, wandering through a life-scale medical encyclopedia of the 18th-19th century frozen in time. The physical layout of the exhibit in the room reinforces this encyclopedic effect. The sections, rows of cabinets, are organized mainly by bodily systems and body parts and medical and scientific fields: starting with an embryonic section, fetal anomalies, gynecological material, followed by the cardiovascular system, the thoracic and abdominal organs, genitalia, the urinary system, shifting to tattooed skin, zoological and comparative anatomy, general anatomy, skeletal system and skeletal injuries and “deformities”, the limbs, the musculoskeletal system, the head, neck, jaw and teeth, the brain and spinal cord, and so it goes. The air feels a little bit stuffy and there is a slightly heavy, enclosed and pressing atmosphere, perhaps because of the lack of windows and the darkness, combined with so much going on inside the displays. There are a few large 2x3m posters against the right wall at every section with an image that pleases my aesthetics senses, one of a palm print, another with some skulls, another showing a digestive system, each a simple white silhouette on light-blue negative space which looks very modern in contrast to the cabinets and their interiors. It helps to relieve the eyes. These posters, along with the clear sharp shapes of the frames and of the general architecture, give the feeling we are peering into the past, from the future. The eclectic mass of the collection, the dead organic material from times past is all contained behind glass, separating us from the contents, for us to look at and learn.

I notice that both the cabinets themselves as well as the room we are in are black rectangles illuminated from above. And I slowly start to feel as if I become part of the exhibit, a performance of “the ill body still alive”: sooner or later parts of me could end up in a cabinet too. I can already picture walking past the reproductive organs section, seeing pieces of my insides in a jar with a little explanation card of a disease.

About halfway through the right-hand side of the exhibit, my stomach begins to feel queasy—I suppose the stuffy smell is getting to me. That and gazing at specimen after specimen of dead human matter—not simply via my computer screen or book (which during my preliminary research I thought had desensitized and prepared me), but in the flesh. This is combined with the practical bombardment of sensory information that comes with examining around two thousand anatomical preparations that the museum says are on display. After an hour, my brain feels jittery at the impossibility of taking it all in, while my gaze jumps from one object to the next.

***

Living with chronic illness has a knack for changing one’s self-concept away from the assumed norm of being healthy and able, towards a familiarity with the realm of sickness, pain, illness and disability (Charmaz, 1983). As a chronically ill audience member and researcher in the Vrolik, I found myself entering the encounter with an urge for relating to those medicalized rather than to the doctors or anatomists doing the medicalizing. Yet at the same time, scientific and medical frameworks of knowledge also feel culturally and epistemologically familiar and authoritative. Thus, these orientations I arrive with are shaped by my own history (Ahmed, 2006), and their potential for contradiction took shape in a recurring embodied response of dissonance. The historical context of the medical museum, the Vrolik’s curation deploying cues that immerse us into Europe’s era of scientific cabinets of curiosities, and the artificiality and strangeness of seeing a prepared piece of a dead body undecomposed, serve as “orientation devices” (Ahmed, 2006) that encourage a medical gaze/approach. They decontextualize the remains as part of a body or person, and recontextualize it into a different narrative. The associated perspective and protagonism is that of doctors, medicine, anatomists, and the large texts on the walls narrating their biographies and careers reinforce this assertion. However, our own experiences as patients, embodied and in the flesh, and the self-awareness of the vulnerability of our bodies and health encourage a different kind of orientation, one of identification or empathy with the material on display as belonging to persons with perspectives of their own.

In reference to a specific “specimen”, a respective info card would state the disease or name the physiology. It would say “osteogenesis imperfecta” or “fetal development”, and my brain kept juxtaposing: “person”. I would look into their dead eyes and be all too aware and confronted with the uneasy feeling that this is someone, was someone, with their own story and experiences. This would be more pronounced the more I could recognize the exterior of the body which I am used to seeing as and associating with personhood. The skin, the eyes and the face were particularly evocative for this, as that is where our eyes are often drawn when we look at other beings. This effect also increased the more “whole” the body piece was, like a hand or an injured foot, or full-sized developed conjoined fetuses, thus becoming cognitively recognizable as being or belonging to *someone*. The more sliced or dismembered, and the deeper we delved into the body and saw pieces outside and disconnected from where they would be in a live body, the less pronounced this awareness was of the piece as “person”, the less I could recognize or identify with “it/them”. Starting from my own vantage point, what I could see/feel is that these specimens, or preparations or objects, are more than just that. More than their physiological or pathological name or definition, more than a trophy, oddity, curiosity, illustration of a technique or craftsmanship, more than an item collected by a mister Vrolik, a mister Bonn, Vesalius or Weber, more than a person or a body, more than dead matter, and more than the being they used to be in life. They are all of these things at once: a multiplicity, with new facets revealing themselves as you move to look at them from different angles.

There is thus a dissonance between the affective orientation I bring into the space due to my own situated-ness and the orientations prompted by the museum’s frames. This led the pieces that made up the museum to be dressed in simultaneous roles: the body as material history, biological organic matter, medicalized anatomy, curiosity, anomaly, work of art, property, possession, commodity, trophy—clashing with the body as person, its identity, agency, and subjectivity. Dead or alive, subject or object, the very nature of the material that made up the exhibit kept on shifting, depending on the narrative context of each piece, their at times contradicting and overlapping stories, and the perspective through which they were told and seen. In the following sections, I employ narratological tools to make sense of how these conflicting frames have come to be and continue to operate, as well as to investigate why the multiplicity of narratives I encountered created an affective dissonance and how that dissonance might be mitigated or bridged.

### 5.2 The Vrolik’s telling of medical history

A pivotal framing to these multiple stories is given by the Vrolik as a mediating context, which affords historical value and meaning to the materials and objects found within. It elevates the epistemic status of the stories it tells as a part of history, based on legitimate sources of material evidence, documentation and physical remains. It facilitates placing its contents as a part of a larger story of evolving medicine and medical knowledge production: “enabl[ing] lumps of brute matter—instruments, wax models, pieces of furniture, anatomical specimens and so forth–to come to life as parts of cultural and social history” (Arnold, 2004, p. 145). In effect, this simultaneously serves to animate/cast the specimens into a particular role as objects of medical history, and to orient us as audience towards looking at them as such, taking on a medical/scientific perspective/gaze. The layout, packed old wooden cabinets, and the aforementioned “time capsule effect” transports us to a context which facilitates this relationship. The “majestic” atmosphere of the museum installation I felt in my visits further served to advance this narrative: eliciting *awe*, triggering *curiosity* and suggesting *wonder* at the scientific feats of our ancestors, upon which current science was built.

This framing capacity can be noticed rather viscerally in light of the contrast experienced while walking around the surrounding corridors outside the exhibit proper, within the university hospital. An eclectic mass of specimens and objects reside around these outer walls. Contrary to the items inside the museum, these pieces do not have spotlights to illuminate them, nor the darkness to protect them from natural light, nor info cards to name or explain what they are. They felt haphazardly put together, with blank patches between them, unlike in the museum where every centimeter of space seemed intentional and used to maximum capacity. They carried an air of being forgotten, while inside the museum walls the air spoke of importance. It was walking along this back wall that I stumbled upon a dead bird, or several, technically speaking. There were the bird skeletons inside the cabinets, important enough to be enclosed but perhaps not enough to be with the other skeletons inside the museum itself. Then there was another bird behind glass that caught my attention. On the pavement, through a window to the outside of the hospital, it lay decomposing with most of its feathers still attached. Seeing the same kind of animal remains facing each other behind their respective glass walls, while some are in glass crypts, and the other is lying without anyone’s notice or interest, brought an affective awareness of the power of these walls to endow matter with meaning and to create hierarchies of meaning within them.

This sight brought my awareness to another aspect that differentiated the bird rotting outside to the remains preserved inside the Vrolik. The heritage function of the museum involves telling a story in which the remains and preparations on display are objects of medical knowledge. They have been turned into objects by the hand of humans, anatomists, “medical men” of the past. The preservation of these specimens is not so much about the individual beings the remains came from, but about the knowledge that scientists of the past could draw from the process and products of their dissection and preparations. It was about the study of the remains, what insights those insides could afford on the general inner workings and structures of the body and disease, how that contributed to medical knowledge at the time, and the skills and adeptness that is proven in elaborating these specimens. The individual as such only mattered in their specificity if they possessed a medical anomaly, so they could be *used to illustrate* said anomaly. The glass (of the hospital, the museum walls and the glass cabinets that hold the specimens) separates ordinary living and dying beings from preserved relics that form part of the history of medicine. By virtue of their being medically objectified, transformed into anatomic specimens, these dead remains are “re-animated” and given a new “post-mortem life” (Alberti and Hallam, 2013) endowed with esteem and importance. In the (hi)story of medicine, they take on a new role: to be seen, stared at, learned from, and evoke emotions of reverence and interest. This renewed animacy is however limited as they are used as vehicles for meaning “bestowed from the outside”, rather than recognizing the “vitality” or “aliveness” they already have (Truman, 2019).

### 5.3 The anatomists as agents and authors

This transformation of “mere matter” into “specimens” is occasioned by the anatomists, their tools and skills. When we enter the museum, along the left walls there are large chunks of text giving us background information about the most pivotal anatomists who contributed to the collection and some historical information surrounding the developments of science and medicine at the time. These texts are not meant to be objects of history themselves; rather they frame the exhibit, written on the very walls that contain it. Similarly, the museum website’s first page retells the story of the museum as originating from the collection of the Vroliks (About the Museum, 2024). These framing texts give the anatomists and medical practitioners ample space and recognition as protagonists of the history of medicine. And the space that is dedicated to them personally gives an impression of high regard and value. They are the acknowledged “contributors”, and it is their identity, legacy and agency that is reaffirmed in the most visible and prominent form. There is extensive information on the website, in the museum brochure and info cards, about the techniques the anatomists used to create the specimens and preparations, thereby enabling new medical knowledge to evolve. They would dissect, slice, color and inject, use substances like alcohol and wax, and suspend pieces in jars. They were often pioneering preparation techniques, advancing scientific knowledge of the body thanks to their power to make the “unseen” *visible*. They would make choices about what to keep of the remains they had to work with and what to dispose of, and so acted as arbiters of value. In these capacities and roles, they are presented as agents and actors, emphasizing their ability to shape and transform matter.

In this transformative process and with the products they create, the anatomists also author stories, whether or not consciously or intentionally so. This is first done in the very procedure of dissection and crafting of specimens. They etch their vision into these bodies, as they inscribe their own meaning and understandings into them. Naming pieces along the way, separating organs from tissue and system. Determining where one anatomic and physiological piece begins and another ends. This procedure is physically both delicate and violent, as it involves the literal breaking and cutting apart of the body. When making corrosion casts for example, the material remains are injected with a hardening material, such as a metal or wax, which fills the cavities of interest to the anatomist. The next step is to get rid of the original organic tissue, to reveal the casted inner structures of which the tissue acts as the mold. This is a destructive process, often done via boiling, maceration, or using acid, enabling the anatomist to wash away the “unwanted” remains (Hendriksen, 2019). This transformation renders the specimen’s original “personhood” less recognizable not only visually but also in their very matter. The violence involved in these processes is meant to be obscured by the new “product”, yet I felt it continue to haunt and linger in the exhibit. By affectively empathizing with the matter on display, my awareness was brought to how the cuts, slices, injections, liquids and so forth distinguish specimens from live bodies such as my own, enabling me to trace the physically transformative processes the pieces have undergone in order to “arrive” and be placed here in front of us.

**Figure 1:**
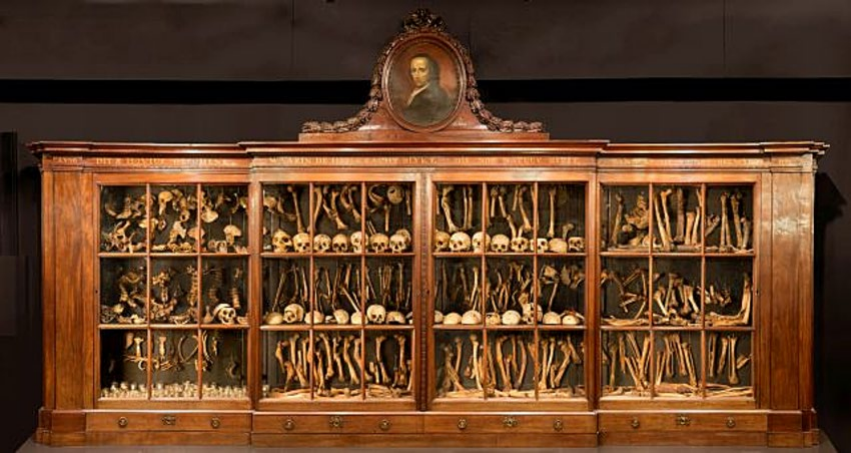
Image of Hovius’s cabinet courtesy of Museum Vrolik (Wiersema, 2020).

There is yet another story layer implicitly present revolving around how the specimens served as possessions, trophies and status-symbols, which can be read particularly clearly in “Hovius’ cabinet of bones” (Figure 1), an important element of the Vrolik’s exhibit. Hovius agreed to donate his collection of bones only if it would get a custom-made cabinet to be kept in to protect them. The bones are mostly anonymous. However, at the very top and center of the adorned cabinet lies a portrait of Hovius himself, a gesture arranged by the professor then minding the collection. The very convictions that led to enshrining a portrait of Hovius, looming over not his own remains, but the skulls and bones he collected, gives testament to how entrenched the notion of prestige and identity were in the practice of collecting and preserving anatomical specimens at the time, providing another layer for their objectification. When presenting a specimen, the people and bodies that they are derived from are no longer recognized, except in occasional records when medical histories were deemed relevant. They were displayed not for the remembrance of the dead, but to further serve medical study as objects: the embodied knowledge of “patients” was not seen as worthy of preservation as their actual bodies. It is likely that these identities and lives were not given importance at the time, since the only bodies that could be legally dissected were those of criminals (often as part of their punishment) or later on those of the impoverished, orphanages, or psychiatric or charity hospitals, unclaimed by family (Ghosh, 2015), as well as bodies of those who were colonized and enslaved at the time (Parry, 2021). This is also evidenced in some of the notes in the Vrolik catalogue, published by Dusseau (1865). Although many entries are indicated as having origins unknown altogether, at times it is mentioned that the bodies were originally of the poor (Dusseau, 1865, e.g., p. 19), or the convicted (e.g., p. 188), or foreign seamen (e.g., p. 29), and a significant portion belong to people of color subjected to European colonial projects (de Rooy, 2023; Dusseau, 1865). Thus, persons who already experienced societal exclusion or oppression were also the ones whose bodies were used in such ways that their identities and personhood would be erased. Instead, as specimens they represented the social, professional and scientific standing and achievement of the new owners towards their wider community.

The anatomists make further use of these specimens to advance new stories such as their theories about evolution, the genesis of a certain illness or fetal development for example, but also more sinister ones, about racial differentiation and phrenology (the study of skulls to determine a person’s character) (About the Museum, 2024; Heiningen, 1997; de Rooy, 2023). Some stories are simply defining what “ill”, “deformed” or “healthy” look like, separating the “normal” from the “pathological”: “for vivid and tangible demonstration of what could go wrong with the body, as well as what a healthy body should look like” (Alberti and Hallam, 2013, p. 6). Hereby, they also authored stories that would reverberate and ripple into social and cultural perceptions of (ab)normality and bodily difference.

### 5.4 Addressing missing and troubled (hi)stories

Despite this historical baggage, there are several ways in which narratives centering “patient’s perspectives” are part of the exhibit. Firstly, by sharing occasional non-medical information related to the body and health which enables the individual specimens to be seen in a socio-cultural context beyond the remit of the medical domain. Some examples include: A snippet on the website that acknowledges the history of keeping remains of saints as relics before the scientifically motivated collecting began in the Renaissance (Techniques, 2024), in the info card of a particular foot that explains an old and abandoned Chinese practice of “foot binding” to create “lotus feet”, or the writing about Hovius’ cabinet of bones that notes that life in the 18th century was different than today’s with the kinds of illnesses, injuries or issues such as malnutrition that impacted many bodies at the time. By acknowledging the cultural, historically contingent and situated dimensions, these bits of information transported me in time and place, not to the medical laboratory or archive, but to sites of everyday life in which people navigated matters of health, the body and illness throughout history. However, these examples are few, leaving me with many critical questions: Who did these remains originally belong to and what are their stories? What would they have felt about pieces of their bodies being here? Would it have been exciting to be preserved for posterity, having a posthumous after-life on a pedestal or in a jar? Or would it have felt like a desecration? Their bodies claimed for reasons beyond them, and used in ways they had no say about.

Although the museum does not provide much material to answer these questions, this is not entirely a choice of omission. Current curators contend with a lack of historical information connected to the pieces in the collection, that which was never gathered, such as details of who they originally belonged to or how exactly they were acquired (de Rooy, 2023). In the cases where we do have this kind of information, we must also struggle with the ethical issue of privacy which is afforded by the anonymity of specimens. Not disclosing names and personal information can be a form of respect to the deceased whose remains are preserved, since the way some of these bodies have been used and are permanently displayed can be deeply invasive. Moreover, forever memorializing their names exclusively in this context has the potential to further reduce their personhood to objects of medical history. At the same time, it can also be seen as humanizing to tell stories of their life in such a way that acknowledges their subjecthood beyond medical objectification. This a significant limitation in the museum’s ability to re-introduce “patients’ perspectives” of the past, therefore further reflection, ethical considerations and research is needed to make informed decisions about what and when to disclose of the persons whose remains are in the Vrolik.

What can be addressed without ethical considerations about the privacy of individuals is the larger historical context within which the remains were gathered, and indeed Museum Vrolik has put effort into acknowledging and researching some of the problems surrounding its preservation and display of human remains. Specifically, in one of its information cards they acknowledge the ethical, moral and legal considerations around how the bodies were acquired at the time were very different from today’s, and that we do not know to what extent consent was requested or given prior to death. The website notes that collections did afford “status” to the medical doctors who gathered them, but asserts that education, research, and now also medical material history, are its main purposes ensuring a respectful context (About the Museum, 2024; Human Remains, 2024). They also have an extensive statement regarding human remains from former colonies of the Netherlands, explaining why they are problematic and how they were used historically by anatomists, some of whom were contributors to their collection, in order to study and argue about their theories on “race”. Hereby they also clarify the relevance of their conscious choice to not display racialized human remains. They further explain how this has a continued legacy of oppression today, and assert their commitment to researching this topic, and to repatriate human remains if this is requested by source communities. This was put into action in 2018 when the Vrolik returned remains to a Māori delegation (Remains of Māori Back in New Zealand, 2024), and continues currently via a partnership with the research project “Pressing Matter” which investigates “Ownership, Value and the Question of Colonial Heritage in Museums” (About the Museum, 2024). This grappling with colonial legacy is unfortunately not an active part of the physical exhibit, but can only explicitly be found on the website and in publications of the current curator (de Rooy, 2023).

However, other forms of historical oppression and marginalization are seldom addressed in the exhibit and would benefit from such conscious engagement. Specifically, the role of ableism is missing considering how the Vrolik is a setting where anonymous individuals with physical differences and/or disabilities are displayed to be stared at and used to give a visual representation of “abnormality” to the public. It orients us—the presumed (able/healthy) viewer—at the center point of reference, relegating the disabled to “some faraway edge of the world” (Garland-Thomson, 2009, p. 42) which we get to meet in a staged encounter that encourages medical objectification with little humanizing context. In the Vrolik’s self-published book *Forces of Form* the massive collection of the Vroliks’ fetuses are described as “wonderous little curiosities preserved in its jars,” that “are keeping science alive” (Oostra, 2009, p. 120), reinforcing concerns that even when an educational context and “respect” is emphasized, this is limited when we do not talk and reckon with our troubled histories and language that sensationalize disability as “other”.

Furthermore, taking the museum as a context of education of history of the body, health and medicine, we are missing not only the voices but also more historical context regarding the other stakeholders involved. Although this is hard to find for specific specimens, Laurens de Rooy, current curator at Museum Vrolik, through a close investigation of the skulls in the collection highlights how “most non-European skulls reflect the (expanding) colonial exploits of the Kingdom of the Netherlands in the first quarter of the nineteenth century, and Gerard’s social position within this colonial network” (de Rooy, 2023, p. 316). He also hypothesizes that the military conflicts in the Northern and Southern Netherlands during the collectors’ lifetimes may also have provided a source for human remains gathered by military doctors working in field hospitals (p. 318). The Vrolik catalogue gives us further insight into how many of these remains were acquired: Directly from burial places, through purchase, or via donations from other anatomists, physicians, collections and from (field) hospitals, especially overseas (Dusseau, 1865). We can also embed the exhibit into a larger European historical socio-cultural context of the collection of human remains for anatomical purposes. Laws needed to emerge to avoid the unethical handling of human remains, such as the practice of grave-robbing which became common in the 14th century, and continued into the 19th century (Ghosh, 2015). Being dissected was historically considered part of criminal punishment, and was often used as a deterrent for crime, which gives insights into how negatively it was viewed by the public for one’s body to be given that fate (Brenna, 2021). There are brief moments when these darker histories are touched upon, for example one info card states that when there was no money for a burial of an orphan child their bodies would be used for science. However this kind of historical legacy is not actively engaged with and seems to receive only anecdotal mention in the exhibit itself. This leads the educational approach and declared sensitivity and respect towards human remains to seem limited in practice.

The most immediate way in which I experienced “patients’ perspectives” to be made present throughout the exhibit involved the Vrolik’s role as a site of education about the human body wherein a physical connection was drawn between the bodies on display and my own. The museum displays the human body and its insides in such a way that we can gaze inside, beyond the boundaries usually provided by the skin and social appropriateness. Through this physical insight and the enabled intimacy, the “objects” can be seen as having an inherent capacity to “invite the viewer to reflect on themselves” (Alberti and Hallam, 2013). The viewer is brought into the matter examined, as we can relate to what we see on the basis of being a body ourselves. As a site for scientific and medical education about the body, the Vrolik actively deploys the potential of its contents to invite the viewers inwards via the information cards provided along with the displays by naming each of the items and then giving the physical context of where it lies anatomically. These specifics enabled me to see the specimens, which at first sight felt eclectic and random, not only for their abstract biological significance but for their relationship to my own body. This effect was more present the more detailed and embodied the information was on the info cards, making direct links between what is on display and the audience’s own body, for example: “see for yourself how your tongue changes in shape and position when pronouncing all of the letters of the alphabet” to explain how the tongue muscles (that you can see in front of you) also feel and function, so you can experience how they matter to your embodied reality. Another example, “when you have a cold the first thing to become inflamed is the mucous membrane of the nasal cavity…one of the symptoms is a throbbing pain on the forehead and left and right of the nose” is the text that illustrates the connection between the nose and sinuses, and how the symptom of that localized pain can point to embodied knowledge of being ill.

These kinds of statements do not only draw the reader in to reflect on themselves, they also assert the epistemic capacity and authority we hold in experiencing our bodies, in a spectrum of health and illness. In these small gestures, we, the audience, are acknowledged as embodied knowers with epistemic agency. This was for me the most effective way that “patients’ perspectives” were made present, where I felt really a part of the exhibit, not as a potential object but as a participant, as a knower, and where my body was explicitly involved in that knowing. It was also at the same time a reminder that its contents are also made up of bodies, just like us, inviting empathy with their past sentience. We are explicitly made aware, as we gaze at an anonymous tongue, of our own tongue, drawing a direct pathway for connection rather than objectification. There is still so much potential for the Vrolik to engage this way with its contents, telling more stories that integrate and protagonize the relationship between the audience and specimens, based on our shared embodied and epistemic agency. This, together with a more active engagement with the existing legacies of the people whose bodies are on display and the historical and political contexts in which the museum’s “specimens” were “produced” would contribute to significantly reducing the affective dissonance I experienced. It would also help others who do not share my particular positionality experience themselves in relation to the people whose remains surround them as agential subjects in the present and history of medicine and illness—opening up perspectives beyond the previously prescribed observer-object dynamic.

## 6 Conclusion

In analyzing my field notes, the most central pattern emerged as a feeling of dissonance. Although the exhibit succeeds in immersing and transporting the audience to learn about a particular time and place in the production of medical and scientific knowledge, when searching for “patients’ perspectives” I often felt at a loss, even though their bodies were right in front of me. I was searching for something I could catch glimpses of at times, but mostly felt in its absence. The remains on display have been decontextualized from their original home as a part of someone, and through the processes of death, dissection, preservation, preparation, and later curation, they became re-contextualised, transformed and “emblazoned” (Sawday, 1995) into specimens in a museum. At the same time, my lived experience and identity as a medicalized person with chronic illness brought an impulse/intensity towards identification and closeness to the “specimens”, grasping for a sense of their agency, voices, perspectives, personhood. From these simultaneous orientations, the remains exist as multiplicity and assemblage, more than who they were in their previous life, and more than what it is presented as today, with new sides revealing themselves at every angle (Ahmed, 2006). These dissonant natures coexist, and cannot be neatly reconciled. What was once human remains is now also an anatomic specimen. Making sense of and grappling with this dissonant multiplicity brings us to a fundamental concern: whether the body is taken as an “object” to gaze at, learn from, act upon; or whether it is seen as an agential subject with perspective of its own. When we are oriented towards the displayed bodies with an objectifying gaze, I am turning to face them as opposed to myself, to be in some way used. When I look at them as potential actors with their own perspectives, I turn not only towards them but I also place myself beside them, and attempt to gaze out at the world from their vantage point, involving a cognitive-emotional act of empathy (even though empathy with the dead involves of an inherent amount of projection and uncertainty). Throughout the research process, it became clear to me that the sense of incongruity I experienced was not merely because of co-existing clashing meanings and orientations, but rather the dominance of medical scientific frames and neglect of “patients perspectives” alongside them. The more I realized the extent of the presence and authority of scientific narratives and absence of the identity and personhood of the remains, the more I felt the affective dissonance magnified.

The neglect of “patients’ perspectives” as another narrative that is curatorially woven into the exhibit led to a sense of dehumanization. My stomach churned not only because I was seeing cut up dead bodies in jars, but also because their “personhood” seemed like a footnote to the exhibit as a whole. How the exhibit is curated serves as a re-enactment of a historically troubled narrative which the Vrolik insufficiently addresses while it tries to distance itself from the unethical acts in its history. Medical frames do not necessitate dehumanization, if patients are understood primarily as persons, and their subjective quality of life, experiences and epistemic authority are given their due importance. Although the museum clearly states their intention of respect and care towards those whose bodies are on display, to shy away from the role dehumanization has played in medical history and to reproduce the asymmetry between the agency and authority of the stakeholders involved reinforces the continued objectification of the remains on display (and the erasure of their former owners’ personhood). We are encouraged to see them as objects of medical knowledge or of medical history rather than to recognize them as (also) persons with perspectives and epistemic authority of their own, not orienting us towards imagining what a story in their own voice might sound like, what seeing through their own eyes may look like, what living in their own bodies may feel like. This dynamic supports both the historic and ongoing epistemic hierarchy between those who study the body, illness, disability, and those who live and experience this first-hand in their own bodies, between those who enact medicine and those whom it is enacted upon.

A first and fundamental step in the direction of making “patients’ perspectives” present can be to start to acknowledge and engage with the multiplicity of possible narratives in medical history, and from there to bring more stories, voices and perspectives into the telling of (hi)stories of health and disability. Specifically, to acknowledge the perspectives of those who have lived with embodied experiences of health, illness and disability, and those who are put on the receiving end of the medical gaze. It also means grappling actively with problematic aspects of the legacy of medical research and medical museums and discussing how this heritage shapes our world today, without yet having all the answers (Majerus, 2017; Parry, 2020; Tybjerg, 2018, 2019; Whiteley et al., 2017). Engaging in this process can be a much stronger statement than trying to reassure visitors that “things are different now” (Birdsall et al., 2015). We can also take other projects as references for dealing with these complex challenges, such as the reinvention of the Anatomical Collection at the University of Jena which was “based on ethical considerations” (Lötzsch and Redies, 2023) and draw on their shared knowledge and experience. Another example is a recent proposal of “*Recommendations for the Management of Legacy Anatomical Collections*” (Cornwall et al., 2024) aiming to centrally address moral and ethical concerns. Furthermore, involving those whose bodies are at stake to have access to shaping the museum setting and bringing in their critical knowledge and perspectives for navigating this murky terrain would serve to both acknowledge their epistemic authority in the matter, but also to avoid unnecessarily taking pieces off display in order to sanitize the exhibit and avoid controversy, as this could lead to a misrepresentation of our problematic collective heritage.

There are many further avenues for exploring the integration of “patients’ perspectives” beyond what has so far been discussed in this research. One very accessible practice is the display of medical instruments and research tools, which have the potential to trigger visceral empathy, depending on surrounding curatorial decisions: “objects also bring to mind the bodies of those they were used upon, and can encourage visitors to project their own bodily experience into either position”, (Whiteley et al., 2017, p. 61) not only the doctors’. Further engaging with other senses than vision, which in this context carries with it the associations with the medical gaze, can also encourage audiences to connect with the exhibit with more embodied and sensorial awareness of their own body and therefore the lived experience of health and illness. An example of this in practice is the use of soundscapes that has been suggested to also bring in literal voices of those previously silenced (Birdsall et al., 2015). In addition, the use of imagination and creative practices which protagonize bodies and patients or narrate from “patients’ perspectives”, hold great potential for creating avenues of empathy and connection, a feeling *with*, rather than the sympathetic and distancing feeling *for*. This can pull from the rich work on narrative illness by thinkers and writers such as Frank (1995), Charon (2006) and Lorde (1997) that have developed extensive hermeneutic tools through which to make sense of illness experiences.

Furthermore, the use of embodied inquiry such as the one exercised in this research project can also serve as an avenue for generating embodied knowledge from more diverse perspectives than those whose stories are so far represented in the exhibit. It can also be a fruitful tool to encourage connection and sensitivity in the audience no matter their positionality and experience. Acknowledging that there is an absence of voices and perspectives, to make an effort to listen to that void making the absence tangible, may serve as a first step in making patients, the ill and medicalized, more present as subjects even in their silence. To move forwards from there, persons with disabilities, illness, bodily differences, impairment and injury, need to be included and recognized in their capacity as knowers, as having vital embodied knowledge via their lived experiencing, as narrators and subjects in the stories that are told. From these stories, we can generate new avenues of understanding health, medicine, illness and disability, of curating and framing museum exhibits, of making sense of our past and present, and of understanding ourselves and each other.

## Data Availability

The original contributions presented in the study are included in the article/supplementary material, further inquiries can be directed to the corresponding author/s.
